# Understanding Patient Beliefs in Using Technology to Manage Diabetes: Path Analysis Model From a National Web-Based Sample

**DOI:** 10.2196/41501

**Published:** 2023-05-03

**Authors:** Karim Zahed, Ranjana Mehta, Madhav Erraguntla, Khalid Qaraqe, Farzan Sasangohar

**Affiliations:** 1 Department of Industrial and Systems Engineering Texas A&M University College Station, TX United States; 2 Texas A&M University in Qatar Doha Qatar; 3 Center for Critical Care Houston Methodist Hospital Houston, TX United States

**Keywords:** type 1 diabetes mellitus, self-management, intention, psychological models, tremor, hypoglycemia, mobile app, health technology

## Abstract

**Background:**

With 425 million individuals globally living with diabetes, it is critical to support the self-management of this life-threatening condition. However, adherence and engagement with existing technologies are inadequate and need further research.

**Objective:**

The objective of our study was to develop an integrated belief model that helps identify the significant constructs in predicting intention to use a diabetes self-management device for the detection of hypoglycemia.

**Methods:**

Adults with type 1 diabetes living in the United States were recruited through Qualtrics to take a web-based questionnaire that assessed their preferences for a device that monitors their tremors and alerts them of the onset of hypoglycemia. As part of this questionnaire, a section focused on eliciting their response to behavioral constructs from the Health Belief Model, Technology Acceptance Model, and others.

**Results:**

A total of 212 eligible participants responded to the Qualtrics survey. Intention to use a device for the self-management of diabetes was well predicted (*R*^2^=0.65; *F*_12,199_=27.19; *P*<.001) by 4 main constructs. The most significant constructs were *perceived usefulness* (β=.33; *P*<.001) and *perceived health threat* (β=.55; *P*<.001) followed by *cues to action* (β=.17; *P*<.001) and a negative effect from *resistance to change* (β=−.19; *P*<.001). Older age (β=.025; *P*<.001) led to an increase in their *perceived health threat*.

**Conclusions:**

For individuals to use such a device, they need to perceive it as useful, perceive diabetes as life-threatening, regularly remember to perform actions to manage their condition, and exhibit less *resistance to change*. The model predicted the intention to use a diabetes self-management device as well, with several constructs found to be significant. This mental modeling approach can be complemented in future work by field-testing with physical prototype devices and assessing their interaction with the device longitudinally.

## Introduction

### Overview

Diabetes is a prevalent condition affecting more than 400 million adults worldwide [1]. To limit serious complications, patients with diabetes need careful adherence to a self-management regimen, which includes monitoring of critical values such as intake of carbohydrates, blood sugar levels, and medication or insulin adherence [2-4] and help patients form healthy habits [5-7]. Several technologies exist to support diabetes self-management, such as blood glucose monitors and continuous glucose monitors. However, despite the promise shown by these technologies, user engagement and satisfaction are relatively low [8]. For example, while the recommended number of blood sugar measurement using blood glucose monitors is 4 to 10 times per day for patients with type 1 diabetes [9], studies show that the majority of patients measure their blood sugar an average of 2-3.5 times a day [10,11].

Several factors may contribute to poor adherence to continuous glucose monitoring use, including discomfort, costs, lack of technological savviness, and overall low interest from the users to sustain engagement with the technology [12,13]. Several behavioral models have emerged to understand contributing factors to such health-related behaviors. One such model, which is commonly used in the literature, is the Health Belief Model (HBM), which provides a framework to explain how an individual’s perceptions (eg, barriers and self-efficacy) influence intention to perform health-related behaviors [14]. Recent work has attempted to integrate constructs from HBM with the Technology Acceptance Model (TAM) [15] to improve the prediction of intention to use patient-facing technologies for hypertension with promising results [16,17].

However, to our knowledge, such models have not been used to understand the intention to use diabetes self-management technologies. In addition, the application of beliefs and acceptance models has mostly focused on an existing technology. Investigating the efficacy of such models during the early phases of design remains a research gap. With the widespread use of mobile health and home telemonitoring technologies [18], understanding the potential impact of beliefs and acceptance on intentions could potentially inform proactive methods to identify variables that form the perception of a particular technology. Since participants’ beliefs regarding their health and acceptance of technology may influence intention and actual usage, knowing the key belief constructs that must be targeted is of particular importance [19]. By identifying which beliefs limit the adoption of a technology, specific design elements (eg, behavior change techniques, motivational messages) may be integrated into the design of this technology to ensure higher chances of sustained usage [20].

### Objectives

The objective of this study was to develop an integrated belief model that helps identify the significant constructs in predicting intention to use a diabetes self-management device for the detection of hypoglycemia. In this paper, we document a survey of a large national sample of patients with type 1 diabetes mellitus to investigate how health beliefs and acceptance constructs influence potential usage of diabetes self-management technologies. This study is part of a larger effort to design a tool to predict the onset of hypoglycemia by monitoring hand tremors. Inspired by Dou et al [17], we developed an integrated model to identify significant constructs by predicting an individual’s intention to use a diabetes self-management technology that helps detect hypoglycemia.

### Background

Our research used a combination of HBM and TAM constructs but did not investigate intentions to use a specific device or technology. Rather, we used a device-agnostic approach where participants were primed to think about how they manage their hypoglycemia and then asked to think about their preferences for a medical device that would help detect the onset of hypoglycemia and manage their diabetes. Accordingly, the following hypotheses were posited.

*Perceived usefulness*, one of the constructs included in TAM, has been shown to influence the use of technology [15]. *Perceived usefulness* refers to how useful and beneficial a system is perceived in achieving a specific purpose. Participants’ prior experiences with or knowledge of diabetes self-management technologies have informed their mental model of such devices [21]. Such mental models include a notion of *perceived usefulness* that may influence adoption. Therefore, we hypothesize that:

- H1: *Perceived usefulness* is positively associated with intention.

The HBM helps identify how certain health beliefs affect an individual’s intention to perform a health-related behavior [14]. *Perceived health threat*, one of the constructs included in HBM, is the extent to which an individual perceives their condition as threatening. Previous literature has suggested that *perceived health threat* has a significant positive effect on perceived usefulness and intention to use [14,17]. Therefore, we hypothesize that:

- H2a: *Perceived health threat* is positively associated with intention.
- H2b: *Perceived health threat* is positively associated with perceived usefulness.

According to HBM, *perceived severity* is one’s opinion of the seriousness and potential impact of their condition on themselves and those around them. While the effects of perceived severity on adherence to new health regimens have shown inconclusive results in 1 study [22], their efficacy in the context of diabetes is worth investigating. Therefore, we hypothesize that:

- H3: *Perceived severity* is positively associated with intention.

*Cues to action*, another construct from HBM, is the stimulus that motivates the adaption of a new behavior. In this study, we refer to *cues to action* as the internal cues and motivations to perform more activity. For example, it has been shown that reminders are effective in improving technology usage [23]. While *cues to action* has not been assessed in relation to intention [24,25], we hypothesize that:

- H4: Cues to action is positively associated with intention.

According to HBM, *perceived barriers* is the perception that challenges exist to performing a healthy behavior [14]. Several barriers have been documented and shown to prevent individuals from adopting new technology as seen in previous literature [15]. Therefore, we hypothesize:

- H5: *Perceived barriers* is negatively associated with intention.

A few behavioral constructs not included in HBM or TAM were also explored. *Past experience* pertains to whether the user has used technology to manage their diabetes. Lack of experience or even a negative experience can reflect a major obstacle for individuals to adopt technology [12]. We hypothesize that:

- H6: Previous positive experience using technology is positively associated with intention.

*Resistance to change*, which is adapted from the dual factor model [26], refers to certain inhibiting beliefs that prevent the undertaking of a new behavior. *Resistance to change* has been found to negatively influence intention to use and perceived usefulness [17]. Therefore, the stronger individuals perceive themselves to be *resistance to change*, the lower their intention to use and perceived usefulness of a device may be [27,28]. We hypothesize that:

- H7a: *Resistance to change* is negatively associated with intention.
- H7b: *Resistance to change* is negatively associated with perceived usefulness.

Finally, the individual’s relationship with their doctor influences how patients manage their conditions. This relationship has been found to significantly predict perceived usefulness of a device [17,29]. Therefore, we hypothesize that:

- H8: Relationship with doctor is positively associated with perceived usefulness.

## Methods

### Participants

A cross-sectional, internet panel survey of 212 adults with type 1 diabetes mellitus residing in the United States was conducted using the Qualtrics platform (Qualtrics) between May and April 2019. The study followed the guidelines of STROBE (Strengthening the Reporting of Observational studies in Epidemiology). The Texas A&M University institutional review board reviewed and approved the study protocol (IRB #2017-0914D) in May 2019 before the survey was launched. Recruitment was arranged by Qualtrics, which has a pool of individuals that can be recruited based on the inclusion criteria provided by our research team. First, a pilot data set consisting of the first 10% (n=20) of responses was shared with the research team to evaluate the inclusion criteria and assess the quality of the response. Individuals who qualified for the survey based on self-reported demographic data (18 years of age and older and diagnosed with type 1 diabetes mellitus) were invited via email to join the panel, with a link to follow if they were interested to participate. Those who participated in the survey were incentivized by points awarded through Qualtrics, which they can later redeem for a reward. Specific logic was added to the instrument to automatically remove unreasonable responses and participants who attempted to answer the questions quicker than a reasonable threshold set by Qualtrics. No identifiable information was recorded, but latitude and longitude were stored by Qualtrics for each respondent and used to confirm that all participants were located within the United States.

### Survey Design

The survey questions were composed such that participants were primed to reflect on how they manage hypoglycemic events and their diabetic condition. As such, the survey targeted three main themes in addition to the demographic information: (1) user perception of hypoglycemia occurrence, (2) diabetes management experience, and (3) the beliefs of users regarding managing diabetes. The focus of this article is on the third theme of the survey, whereas details regarding the other 2 themes of the study are reported elsewhere [11]. The questions (Table 1) were published on Qualtrics and were rated by the participants on a 10-point Likert scale where 1=strongly disagree, 5=neutral, and 10=strongly agree.

**Table 1:** Questions and constructs asked in the survey.

Construct		Question	Reference	
**Perceived usefulness**				[19,22]
	PU1	Logging or sending my blood glucose values would help me manage diabetes better		
	PU2	Overall, a diabetes management technology would be useful		
	PU3	I don't think any device can help me in managing my condition		
**Intention to use**				[19,22]
	ITU1	Given the opportunity, I would like to use a technology that helps me manage my diabetes		
	ITU2	I would consider continuously using such a device		
	ITU3	I am very determined to manage my diabetes		
**Perceived health threat**				[19,21]
	PHT1	I am very knowledgeable of the severity of my diabetes condition		
	PHT2	I am concerned about my diabetes		
	PHT3	I put in effort to manage my diabetes		
	PHT4	I feel keeping track of my glucose levels is very important		
**Perceived severity**				[14]
	PS1	Having diabetes limits my overall quality of life		
	PS2	Having diabetes negatively impacts my job performance		
**Self-efficacy**				[19,30]
	SE1	I am confident in my ability to manage diabetes		
	SE2	If I try enough, I know I can have proper control over my condition		
**Social influence**				[19,30]
	SI1	People important to me think that I should use technology to help manage my diabetes		
	SI2	People who are important to me use a diabetes management tool		
**User experience**				Newly developed
	UE1	I use smartphones to help me manage my condition		
	UE2	My past experience with Diabetes management tools has been positive		
	UE3	I think of myself as a tech savvy person (someone comfortable learning and using technology)		
**Resistance to change**				[19,31]
	RTC1	I do not want the technology to change the way I deal with diabetes		
	RTC2	I do not want the technology to change the way I interact with other people		
	RTC3	I am comfortable with using a diabetes management technology to help me with my condition		
**Relationship with doctor**				[19,32]
	RWD1	Doctors are my most trusted source of health information		
	RWD2	When I have a health concern, my first step is to contact a doctor		
	RWD3	I trust the health care system		
**Cues to action**				[14]
	CTA1	I have heard good things about diabetes management technology		
	CTA2	I know where to go to get my blood sugar history monitored		
	CTA3	I know that I should use technology to help me manage my condition		
**Perceived barriers**				[14]
	PB1	There are barriers to me managing my condition		
	PB2	I am aware of why I am unable to properly manage my condition		

### Analysis

Partial least square path modeling [33] was used to assess the magnitude and significance of the causal relationships between the various constructs similar to the approach in [17]. It was performed to evaluate the hypothesized relationships between intention to use and behavioral constructs.

## Results

### Demographics

All 212 respondents were located within the United States, with 40 out of the 50 states represented in the sample; 129 out of 212 participants (61%) were females, and about half (n=117, 55%) were between the ages of 30 and 50 years, which comprised half the sample size. The data underrepresent older adults who might not be inclined to take a web-based survey and overrepresent the middle age groups. As shown in Table 2, other demographic factors align with the national data available. Most participants (n=182, 82%) were White non-Hispanic, and 92 (57%) participants had a household income greater than US $50,000. When asked if they used technology to manage their diabetes, most respondents (n=150, 71%) indicated that they currently use or have used at least one in the past. While these categories are not mutually exclusive, 41 out of 150 participants (27%) have used a continuous glucose monitor, 49 out of 150 (33%) have used an insulin pump, 107 out of 150 (71%) have used a blood glucose meter, and 57 out of 150 (38%) have used a smartphone app to aid in the self-management of diabetes. Further details on demographic information can be found in [11].

**Table 2:** Demographics.

		Web-based data sample		National data		
Characteristic		Participants, n (%)		Population (%)	References	
**Gender**						[34]
	Female	129 (60.9)		51		
	Male	83 (39.1)		49		
**Age (years)**						[35]
	18-29^a^	34 (16.0)		18.4		
	30-39	64 (30.2)		17.8		
	40-49	53 (25.0)		16.6		
	50-59	33 (15.6)		17.4		
	≥60	28 (13.2)		29.8		
**Race**						[36]
	White	182 (85.9)		76.5		
	Native Hawaiian or Pacific Islander	2 (0.9)		0.2		
	Black or African American	13 (6.1)		13.4		
	Asian	6 (2.8)		5.9		
	Two or more races	6 (2.8)		2.7		
	Other	3 (1.4)		—^b^		
	White non-Hispanic	174 (82.1)		60.4		
	Hispanic or Latino	17 (8.0)		18.3		
**Smartphone**						[37]
	None	15 (7.1)		19		
	Yes	197 (92.9)		81		
	Android	103 (52.2)		51.1		
	iOS	93 (47.2)		48.1		
	Other	1 (0.5)		0.8		
**Income level (US $)**						[36]
	<$20,000^c^		24 (11.3)	19.1		
	$20,000-$29,999^d^		20 (9.4)	8.8		
	$30,000-$39,999^e^		23 (10.9)	12		
	$40,000-$49,999		17 (8.0)	N/A^f^		
	$50,000-$59,999^g^		29 (13.7)	17.2		
	>$60,000^h^		92 (43.4)	42.9		
	Did not answer		7 (33)	—		
**Educational level**						[30]
	None		—	1.4		
	Less than high school		2 (0.9)	4.2		
	High school		36 (17.0)	34.9		
	Some college, no degree		43 (20.3)	21		
	Bachelor's		61 (28.8)	18.8		
	Associate degree or trade school		20 (9.4)	8.2		
	Graduate or professional		50 (236)	11.5		
**Years of living with diabetes**				N/A		N/A
	≤1		69 (32.5)			
	>1 and ≤10		46 (21.7)			
	>10 and ≤25		39 (18.4)			
	>25		58 (27.4)			
**Daily blood glucose measurements^i^**				N/A		N/A
	0		12 (5.9)			
	1-3		85 (41.7)			
	4-10		107 (52.5)			

^a^National data represents those aged 20-29 years.

^b^Data not available.

^c^National data represents income levels <US $25,000.

^d^National data represents income levels from US $25,000 to US $35,000.

^e^National data represents income levels from US $35,000 to US $50,000.

^f^N/A: not applicable.

^g^National data represents income levels from US $50,000 to US $75,000.

^h^National data represents income levels >US $75,000.

^i^Eight entries were removed due to invalid numbers or text.

### Survey Reliability

The average responses for each question are listed in Table 3, along with the reliability metrics. For constructs having 3 or more questions, Cronbach alpha (α>.7) showed good reliability for *intention*, *perceived health threat*, *past experience*, *relationship with doctor*, and *cues to action*. However, Cronbach alpha was lower (α>.5) for *perceived usefulness* and *resistance to change*. Among the constructs that had 2 questions, we witnessed a strong Spearman’s correlation (ρ>0.7) for *perceived severity* and *self-efficacy* which was significant at *P*<.001. A medium correlation (ρ>0.5) was found for both *social influence* and *perceived barriers*, also significant at *P*<.001.

**Table 3:** Response to the belief questions.

Construct			Question		Mean (SD)		Correlation (α)	
**Perceived usefulness**								.5
	PU1	Logging or sending my blood glucose values would help me manage diabetes better		6.79 (2.67)				
	PU2	Overall, a diabetes management technology would be useful		7.62 (2.24)				
	PU3	I don't think any device can help me in managing my condition		4.27 (2.92)				
**Intention to use**								.83
	ITU1	Given the opportunity, I would like to use a technology that helps me manage my diabetes		7.56 (2.49)				
	ITU2	I would consider continuously using such a device		7.57 (2.37)				
	ITU3	I am very determined to manage my diabetes		8.07 (2.02)				
**Perceived health threat**								.71
	PHT1	I am very knowledgeable of the severity of my diabetes condition		8.18 (1.97)				
	PHT2	I am concerned about my diabetes		7.25 (2.46)				
	PHT3	I put in effort to manage my diabetes		7.96 (1.97)				
	PHT4	I feel keeping track of my glucose levels is very important		8.01 (1.98)				
**Perceived severity**								
	PS1	Having diabetes limits my overall quality of life		6.35 (2.91)				
	PS2	Having diabetes negatively impacts my job performance		5.43 (3.12)				
**Self-efficacy**								.70
	SE1	I am confident in my ability to manage diabetes		7.32 (2.21)				
	SE2	If I try enough, I know I can have proper control over my condition		7.65 (2.10)				
**Social influence**								.53
	SI1	People important to me think that I should use technology to help manage my diabetes		6.56 (2.89)				
	SI2	People who are important to me use a diabetes management tool		5.26 (3.30)				
**User experience**								.73
	UE1	I use smartphones to help me manage my condition		5.20 (3.37)				
	UE2	My past experience with Diabetes management tools has been positive		6.53 (2.65)				
	UE3	I think of myself as a tech savvy person (comfortable learning and using technology)		7.02 (2.77)				
**Resistance to change**								.53
	RTC1	I do not want the technology to change the way I deal with diabetes		4.78 (2.93)				
	RTC2	I do not want the technology to change the way I interact with other people		6.52 (3.02)				
	RTC3	I am comfortable with using a diabetes management technology to help me with my condition		7.59 (2.32)				
**Relationship with doctor**								.84
	RWD1	Doctors are my most trusted source of health information		7.35 (2.36)				
	RWD2	When I have a health concern, my first step is to contact a doctor		7.25 (2.39)				
	RWD3	I trust the health care system		6.83 (2.41)				
**Cues to action**								.83
	CTA1	I have heard good things about diabetes management technology		7.08 (2.52)				
	CTA2	I know where to go to get my blood sugar history monitored		7.26 (2.64)				
	CTA3	I know that I should use technology to help me manage my condition		7.02 (2.70)				
**Perceived barriers**								.57
	PB1	There are barriers to me managing my condition		5.70 (2.86)				
	PB2	I am aware of why I am unable to properly manage my condition		6.32 (2.86)				

### Path Analysis

**Figure 1:**
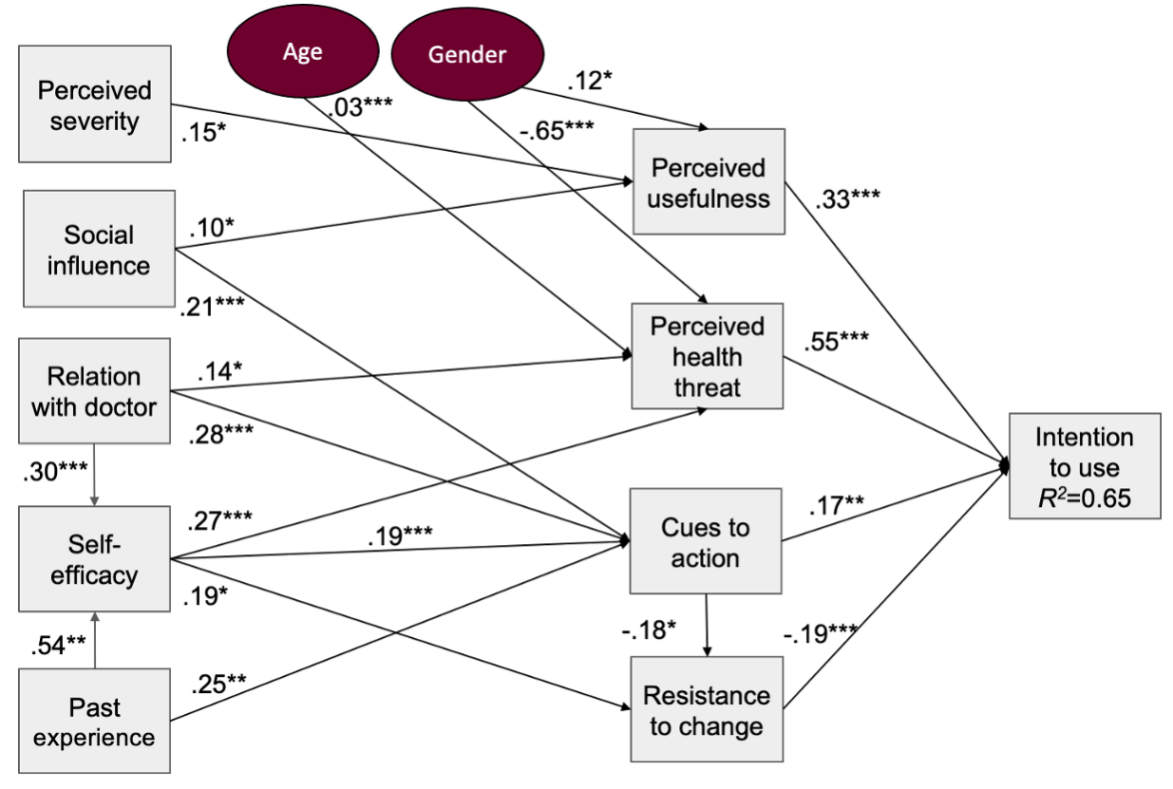
Path analysis model. *P<.05, **P<.01, ***P<.001.

The model was assessed by checking the significance of path coefficients (β) among the independent variables and the latent variables. The results of the path modeling are shown in Figure 1. Each construct was regressed against the other constructs to confirm existing relationships hypothesized above and uncover any relationships that were not accounted for. The model shows that intention to use is significantly influenced (*R*^2^=0.627; *F*_12,199_=27.19; *P*<.001) by *perceived usefulness*, *perceived health threat*, *cues to action*, and *resistance to change*.

Overall, the more useful participants perceived a diabetes self-management technology would be, the more likely they were to use it (β=.33; *t*_199_=4.69; *P*<.001), which supports H1. Male participants were more likely to have a positive perception of usefulness (β=.12; *t*_199_=2.24; *P*=.03). Also, the more threatening participants perceived their condition to be, the more likely they were to intend to use a device (β=.55; *t*_199_=7.02; *P*<.001), which supports H2a. However, *perceived usefulness* was not found to be significantly predicted by a higher *perceived health threat*, so H2b was not supported. Rather, *perceived usefulness* was influenced by *perceived severity* of the condition (β=.15; *t*_199_=2.19; *P*=.03), which is related to *perceived health threat*. Male participants were less likely to perceive their condition as threatening (β=−.65; *t*_199_=−3.81; *P*<.001) and older individuals had higher *perceived health threat* (β=.025, *t*_199_=4.29, *P*<.001). *Perceived severity* did not have a direct effect on intention; thus, H3 was not supported. In addition, a stronger *relationship with their doctor* and stronger *self-efficacy* reflected an increase in *perceived health threat* (β=.14; *t*_199_=2.09; *P*=.03 and β=.27; *t*_199_=5.12; *P*<.001, respectively) so they indirectly influenced intention.

Cues to action positively influenced intention to use (β=.17; *t*_199_=2.73; *P*=.007), thereby supporting H4. Meanwhile, *perceived barriers* did not have any significant direct or indirect effect on *intention*, so H5 was not supported. *Past experience* had a significant effect on *self-efficacy* (β=.54; *t*_199_=6.72; *P*<.01); however, it did not have a direct influence on intention to use, so H6 was partially supported. Also, *resistance to change* had a negative effect on intention to use (β=−.19; *t*_199_=−3.61; *P*<.001), which supports 7a and having high *self-efficacy* made individuals more *resistant to change* (β=.19; *t*_199_=2.25; *P*=.03). However, no significant relationship was found between *resistance to change* and *perceived usefulness*, so H7b was not supported. Finally, participants’ *relationship with their doctor* did not have a significant relationship with *perceived usefulness*, so H8 was not supported. A summary of the hypotheses and their status is provided in Table 4.

**Table 4:** Summary of the hypotheses and whether they were supported.

Hypothesis	Result
H1: Perceived usefulness is positively associated with intention	Supported
H2a: Perceived health threat is positively associated with intention	Supported
H2b: Perceived health threat is positively associated with perceived usefulness	Not supported
H3: Perceived severity is positively associated with intention	Not supported
H4: Cues to action is positively associated with intention	Supported
H5: Perceived barriers is negatively associated with intention	Not supported
H6: Positive past experience is positively associated with intention	Partially supported
H7a: Resistance to change is negatively associated with intention	Supported
H7b: Resistance to change is negatively associated with perceived usefulness	Not supported
H8: Relationship with doctor is positively associated with perceived usefulness	Not supported

## Discussion

### Principal Findings

In this study, we integrated HBM with the TAM and incorporated additional constructs (in line with [17]) to predict the intention of patients with type 1 diabetes to use technology to manage their condition. To our knowledge, this is the first attempt to use behavioral constructs to investigate patient intentions to use diabetes self-management technologies. The findings presented here highlight several significant relationships that may inform future proactive approaches in understanding the adoption and sustained usage of diabetes technologies. In particular, constructs with significant effects on intention may be subject to further investigation to assess their use in behavior change efforts.

The results show that the strongest relationship was between an individual’s *intention* to use technology and the *perceived usefulness* of such technology. This finding supports the premise of TAM [15] and a large body of literature that have used it. The evidence documented here may suggest that adoption and sustained usage of diabetes technologies may depend on patients’ buy-in and conviction about the benefits provided by the technology. For participants to find benefit in such a technology, it must integrate high information quality, personalization, and usable core functions such as notifications, goal setting, and feedback into its design [31]. For example, high-quality content from authoritative sources (eg, American Diabetes Association) may be used to gain users’ trust in the credibility of the content [32]. Personalization may be achieved by including the name of the user while interacting with them and forming a user profile and accounting for their personality [38]. Users must also be educated on the benefits of the technology and provided some form of social proof from other individuals who have used it and benefitted from it [39]. Training users on the technology may help users feel confident they are able to use the device [40]. Ultimately, participants also need to witness an improvement in their health outcomes to perceive it as useful.

Next, *intention* was significantly influenced by *perceived health threat*. In other words, the more serious the condition was perceived to be, the more likely the higher the intention to use a technology to manage it. This finding is in line with other studies investigating eHealth services [41] or hypertension management technologies [17]. Given the importance of this construct for potential impacting behaviors related to adoption and sustained usage of diabetes technologies, future efforts may focus on educational content, reminders and alerts, and information visualization techniques that make the risks that diabetes poses to a patient’s health as well as the consequence of unhealthy behaviors more tangible. For example, descriptive statistics from authoritative and credible sources could be used to make the health threat more salient by highlighting the risks for the patients’ respective demographic (eg, age, race, and location) and long-term complications of not managing their condition [42]. Special care must be given to elderly populations as older age was shown to increase an individual’s *perceived* *health threat*; however, despite high intentions to use, low tech savviness or literacy have been shown to be a major barrier to sustained usage in other studies [43,44].

In addition, *resistance to change* had a significant negative effect on intention to use. Understanding why users are resistant to changing their behavior is important but challenging and due to contentment with their current habit or because there is a certain level of anxiety from trying out a new behavior or technology [45]. Progressive persuasion has been posited as an approach to work around participants’ *resistance to change* their behavior and use a new technology. This method may be implemented by assuring resistant individuals that little change is required, stressing the ease of use, then introducing them slowly to more features over time [45]. Addressing *perceived barriers* may also aid participants to be less resistant to change, which could be a key to help individuals become more willing to engage in behaviors that manage their health [46].

Finally, *cues to action* had a significant impact on intentions. Individuals who often recall performing behaviors related to their regimen, also known as internal *cues to action*, are more likely to use the technology. Individuals who form good prospective memory have more strongly internalized cues and are more likely to remember to perform certain behaviors (eg, measure blood sugar) [47]. Regular reminders and making patients more aware of the need to manage their diabetes could contribute to users forming the intention to use a device for that purpose and, ultimately, establish a habit from this behavior [48]. In fact, reminders have been reported to be among the core functions that an app must have in order to achieve adequate functionality [31]. *Cues to action* is an important factor but has not been assessed for how it is influenced by a longitudinal intervention. Studies may benefit from using automated reminders in an app and testing the change in *cues to action* for those who receive automated reminders versus those who do not.

### Limitations

This study had several noteworthy limitations that may affect the generalizability of the findings. First, our participants self-reported to have type 1 diabetes, and while the sample was drawn from a panel defined by this condition, the research team had no way of validating this claim. Second, the sample’s average age was biased toward young to middle-aged participants, and more work is needed to assess such relationships for older populations. Third, this work elicited at one point in time similar to other studies focusing on beliefs [19,20,27]. Future work should assess longitudinal changes in beliefs and potentially compare intentions with actual usage.

### Conclusion

Proactive and predictive approaches in understanding technology adoption and usage remain a research gap. Behavioral constructs such as health beliefs and technology acceptance show promise in providing potentially useful insight on behaviors. This research showed that *perceived health threat*, *perceived usefulness*, *cues to action*, and *resistance to change* might possess such predictive efficacy in the context of diabetes technology usage. The findings presented here suggest that future work can benefit from the assessment of belief constructs early in the technology design and development cycle to inform areas of opportunity to address bottlenecks and to identify personalized behavior change interventions [49]. For example, a patient scoring low on *perceived health threat* can receive educational messages to increase their knowledge of chronic diseases and their risks, whereas those with high scores on *resistance to change* can receive persuasive and motivational messages. However, work is needed to validate these findings under varied health contexts and with specific technologies.

## Abbreviations

HBM: Health Belief Model
TAM: Technology Acceptance Model
